# Transcriptional Profiling of Advanced Urothelial Cancer Predicts Prognosis and Response to Immunotherapy

**DOI:** 10.3390/ijms21051850

**Published:** 2020-03-08

**Authors:** Seung-Woo Baek, In-Hwan Jang, Seon-Kyu Kim, Jong-Kil Nam, Sun-Hee Leem, In-Sun Chu

**Affiliations:** 1 Genome Editing Research Center, Korea Research Institute of Bioscience and Biotechnology (KRIBB), Daejeon 34141, Korea; baek@kribb.re.kr (S.-W.B.); jangih@kribb.re.kr (I.-H.J.); 2 Department of Bioinformatics, KRIBB School of Bioscience, Korea University of Science and Technology, Daejeon 34113, Korea; seonkyu@kribb.re.kr; 3 Personalized Genomic Medicine Research Center, KRIBB, Daejeon 34141, Korea; 4 Department of Urology, Research Institute for Convergence of Biochemical Science and Technology, Pusan National University Yangsan Hospital, Yangsan 50612, Korea; tuff-kil@hanmail.net; 5 Department of Biological Science, Dong-A University, Busan 49315, Korea; shleem@dau.ac.kr

**Keywords:** bladder cancer, immune checkpoint inhibitor, CD8^+^ T effector cells

## Abstract

Recent investigations reported that some subtypes from the Lund or The Cancer Genome Atlas (TCGA) classifications were most responsive to PD-L1 inhibitor treatment. However, the association between previously reported subtypes and immune checkpoint inhibitor (ICI) therapy responsiveness has been insufficiently explored. Despite these contributions, the ability to predict the clinical applicability of immune checkpoint inhibitor therapy in patients remains a major challenge. Here, we aimed to re-classify distinct subtypes focusing on ICI responsiveness using gene expression profiling in the IMvigor 210 cohort (*n* = 298). Based on the hierarchical clustering analysis, we divided advanced urothelial cancer patients into three subgroups. To confirm a prognostic impact, we performed survival analysis and estimated the prognostic value in the IMvigor 210 and TCGA cohort. The activation of CD8^+^ T effector cells was common for patients of classes 2 and 3 in the TCGA and IMvigor 210 cohort. Survival analysis showed that patients of class 3 in the TCGA cohort had a poor prognosis, while patients of class 3 showed considerably prolonged survival in the IMvigor 210 cohort. One of the distinct characteristics of patients in class 3 is the inactivation of the TGFβ and YAP/TAZ pathways and activation of the cell cycle and DNA replication and DNA damage (DDR). Based on our identified transcriptional patterns and the clinical outcomes of advanced urothelial cancer patients, we constructed a schematic summary. When comparing clinical and transcriptome data, patients with downregulation of the TGFβ and YAP/TAZ pathways and upregulation of the cell cycle and DDR may be more responsive to ICI therapy.

## 1. Introduction

Bladder cancer is the sixth most common malignant disease. In 2019, 80,470 new cases of bladder cancer were diagnosed, and 17,670 deaths were due to bladder cancer in the United States [1]. Bladder cancer is generally divided into pathological subtypes: non-muscle-invasive bladder cancer (NMIBC) and muscle-invasive bladder cancer (MIBC). Cisplatin-based chemotherapy followed by radical cystectomy is the standard of care in previously untreated patients with MIBC. However, patients who relapse after cisplatin-based chemotherapy experience a very poor prognosis [2]. Since cisplatin-based chemotherapy also has the limitation of drug resistance, it is necessary to provide a variety of treatments such as immunotherapy. Previous investigations of immunotherapy have opened a new frontier in the treatment of advanced urothelial cancer [3,4]. Although the response rates are moderately high, it is promising that responsive patients experience durable disease management. Unlike conventional cisplatin-based chemotherapy, immunotherapy enhances the patient’s own immune environment and can be combined with conventional therapies to produce additive effects [5].

Recently, immunotherapy studies have focused on a way to improve efficacy in individual patients. Numerous studies reported several molecular subtypes in bladder cancer, including immunotherapy-associated subgroups, the genomically unstable (GU) subtype of the Lund classification and the neuronal subtype in The Cancer Genome Atlas (TCGA) classification [6,7,8,9,10]. PD-L1 protein expression on immune cells, tumor mutation burden (TMB), and the TGFβ pathway have been shown to correlate with the clinical outcome of immune checkpoint inhibitor (ICI) therapy for advanced urothelial cancer [11]. The previous molecular subtypes of advanced urothelial cancer are not a classification system directly related to immunotherapy. In the existing classification systems, only some patients with specific subtypes of the Lund and TCGA were identified to be responsive to immunotherapy. Furthermore, overall immune system activities such as transcriptional activities of CD8^+^ T effector (T_eff_) cell have not been fully elucidated in bladder cancer. To provide ICI therapy to more patients with advanced urothelial cancer, we wanted to explore both various clinical outcomes and a new subset related to ICI therapy that contains many limiting factors.

In this study, we identified a gene expression signature from the IMvigor 210 cohort [11] revealing distinct three molecular subgroups showing different clinical characteristics and core biological pathways in advanced urothelial cancer patients. To validate a signature, we applied the signature into the TCGA and Lund cohorts and confirmed similar characteristics. By performing a survival analysis, we confirmed that the patients who could potentially benefit from anti-PD-L1 treatment actually represented a difference in the IMvigor 210 and TCGA cohorts.

## 2. Results

### 2.1. Discovery of Distinct Three Subtypes and Clinical Characteristics

**Figure 1:**
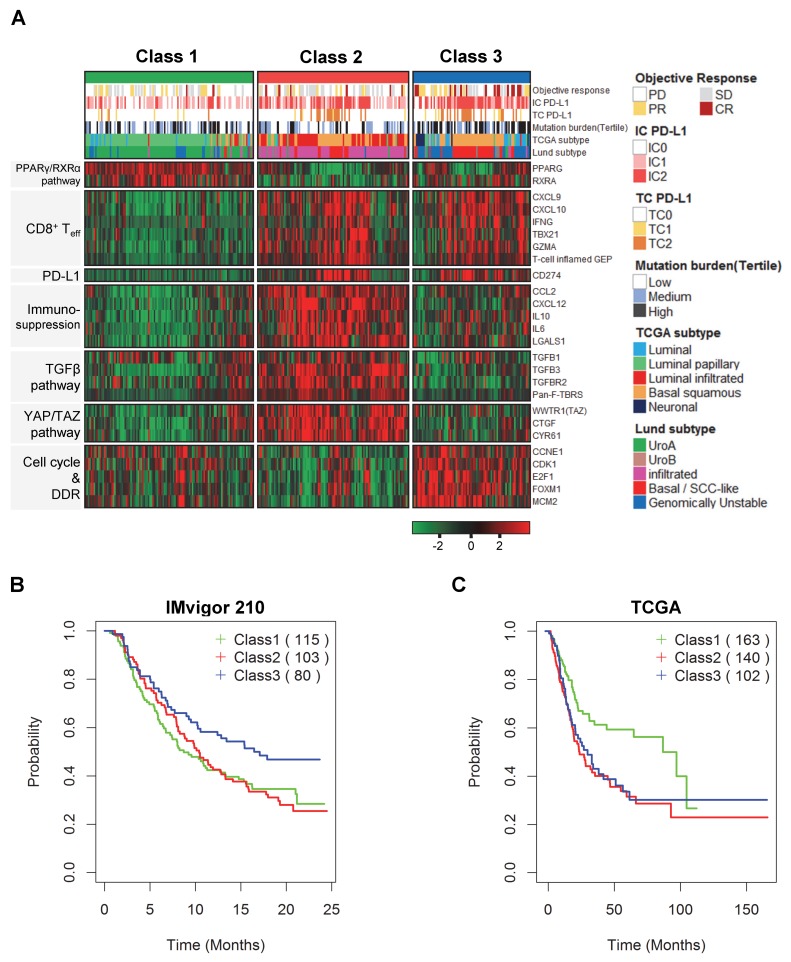
Core biological pathways associated with immune checkpoint inhibitor (ICI) therapy in the IMvigor 210 cohort and survival analyses. (**A**) Heat map of immunotherapy-associated clinical and biological features. **On top**, samples are ordered according to gene expression patterns. Gene signatures, including the pan-fibroblast TGFβ response signature (pan-F-TBRS) and the T cell inflamed gene expression profile (GEP) scores, were selected to explore the correlation between expression patterns and other relevant biological processes. Gene expression levels and signatures such as the Pan-F-TBRS and GEP were ordered and grouped by pathway. The coloring in the heat map reflects relatively high (red) and low (green) expression (Z score) levels; the same representation is used for high and low gene signatures. T_eff_, T effector. (**B**) Overall survival in the IMvigor 210 cohort (*p* = 0.04 by the log-rank test). (**C**) Overall survival in the TCGA cohort (*p* = 0.001 by the log-rank test).

To select patients with a high response to ICI therapy, we performed unsupervised clustering analysis using gene expression profiling from bladder cancer patients treated with the PD-L1 inhibitor atezolizumab (the IMvigor 210 trial [11]). Based on the hierarchical clustering analysis of the expression patterns of 2366 genes correlated with the IMvigor 210 cohort, we then divided advanced urothelial cancer patients into three subgroups (Figure S1). From the clustering analysis results, we chose 24 genes associated with the three subgroups and identified a transcriptional pattern according to these genes. In addition, we identified clinical characteristics such as PD-L1 expression on immune cells (IC PD-L1), PD-L1 expression on tumor cells (TC PD-L1), tumor mutation burden (TMB), and Lund and TCGA subtypes related with the three subgroups. We identified that IC PD-L1 and TC PD-L1 were increased in classes 2 and 3. Furthermore, we identified that TMB was highest in class 3. Importantly, we confirmed that many patients with the neuronal subtype from the TCGA classification were included in class 3 and also a subset of patients with the GU subtype from the Lund classification was involved in class 3 (Figure 1A).

### 2.2. Biological Insight into the Newly Identified Subtypes

Next, we investigated core biological pathways that are known to play major roles in the immune system. Immune cell infiltration is controlled by activated PPARγ/RXRα, which inhibits the host immune response by suppressing the expression and secretion of inflammatory cytokines [12]. The genes involved in the PPARγ/RXRα pathway (e.g., *PPARG* and *RXRA*) were upregulated in class 1. According to a recent report, urothelial cancer patients with *FGFR3* mutations had lower immune cell infiltration and lower TGFβ signals than patients without *FGFR3* mutations [13]. We identified that *FGFR3* mutations were enriched in class 1 (Figure S2). On the other hand, the expression of CD8^+^ T effector cell-related genes (e.g., *CXCL9, CXCL10, IFNG, TBX21,* and *GZMA*) and PD-L1 (*CD274*) was relatively upregulated in classes 2 and 3. In addition, the T-cell-inflamed gene expression profile (GEP), which was correlated with a clinical benefit in a clinical study of pembrolizumab [14], was activated in classes 2 and 3. Exceptionally, in patients with the neuronal subtype from the TCGA classification and some of the GU subtype from the Lund classification in class 3, CD8^+^ T effector cell-related genes and the GEP were relatively downregulated. The expression of immune-suppression-related genes (e.g., *CCL2*, *CXCL12*, *IL10*, *IL6*, and *LGALS1*) was also upregulated in class 2. Furthermore, TGFβ pathway genes (e.g., *TGFB1*, *TGFB3*, and *TGFBR2*) and pan-fibroblast TGFβ response signature (pan-F-TBRS) scores were upregulated in class 2 but downregulated in class 3, consistent with a previous report that TGFβ attenuates the response to PD-L1 inhibitors [11]. Multiple cancer-associated signaling networks engage in regulatory crosstalk with the YAP/TAZ pathway, which has been reported to functionally interact with the TGFβ pathway [15]. The activation of YAP/TAZ-pathway-related genes (e.g., *WWTR1*, *CTGF*, and *CYR61*) also supported the activation of the TGFβ pathway and immune suppression in class 2. Notably, *CTGF,* a major target gene of the YAP/TAZ pathway that is associated with angiogenesis, epithelial-mesenchymal transition and wound healing, was differentially expressed between classes 2 and 3. Additionally, the cell cycle and DNA replication and DNA damage (DDR) genes (e.g., *CCNE1*, *CDK1*, *E2F1*, *FOXM1*, and *MCM2*) were upregulated in class 3. These results support significant differences in clinical characteristics and core biological pathways between the three subtypes.

### 2.3. Prognostic Impact Based on Unsupervised Clustering Analysis

To investigate the utility of the three molecular subtypes, we performed survival analysis and estimated the prognostic value by a log-rank test. As a result, we identified that patients with activated CD8^+^ T effector cells in classes 2 and 3 showed slightly prolonged survival after treatment with the PD-L1 inhibitor. Importantly, patients in class 3 had better survival than those in the other subgroups (Figure 1B). Interestingly, patients in classes 2 and 3 had poorer prognoses than those in class 1 before treatment with the PD-L1 inhibitor in the TCGA cohort (Figure S2 and Figure 1C). These results suggest that patients with poor prognoses in class 3 exhibited prolonged survival after treatment with a PD-L1 inhibitor.

### 2.4. Comparison of Clinical Outcomes in the Three Subgroups

**Figure 2:**
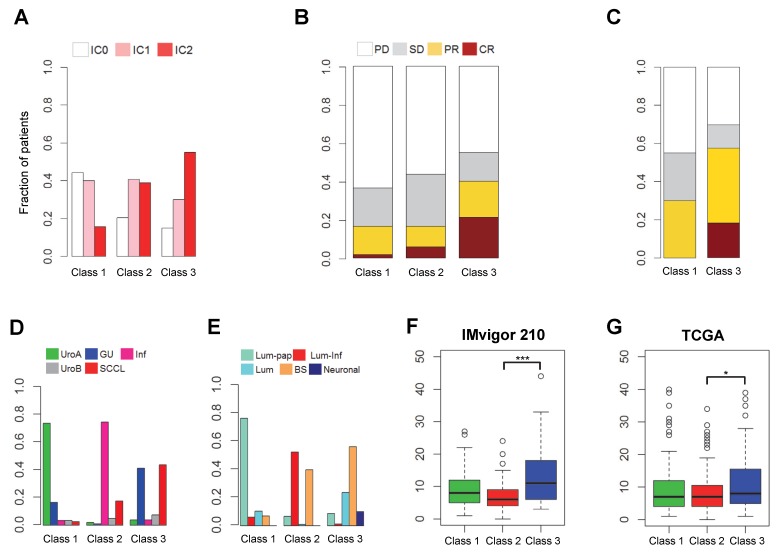
Clinical and biological characteristics of Figure 1A. (**A**) Distribution of PD-L1 protein expression levels on immune cells in each class (*p* = 0.0003 by the chi-squared test). (**B**) Objective response rate stratified by the three subgroups (*p* = 2.714 × 10^−5^ by the chi-squared test). (**C**) Comparison of the objective response rate between class 1 and class 3 in the GU subtype of the Lund classification (*p* = 0.046 by the two-tailed Fisher’s exact test). PD, progressive disease; SD, stable disease; PR, partial response; CR, complete response. (**D**) Distribution of subtypes of the Lund classification in each subgroup (*p* < 2.2 × 10^−16^ by the chi-squared test). UroA, urothelial-like A; GU, genomically unstable; Inf, infiltrated; UroB, urothelial-like B; SCCL, squamous cell carcinoma-like. (**E**) Distribution of subtypes of the TCGA classification in each subgroup (*p* < 4.6 × 10^−51^ by the two-tailed Fisher’s exact test). Lum-pap, luminal-papillary; Lum-inf, luminal-infiltrated; Lum, luminal; BS, basal squamous. (**F**) Reported tumor mutation burden (TMB) classified by the three subgroups (*p* = 1.83 × 10^−8^ by the two-sample *t*-test). (**G**) Reported TMB, classified by the three subgroups in the TCGA cohort (*p* = 0.012 by the two-sample *t*-test; class 2 vs. class 3). * *p* < 0.05, *** *p* < 0.001. Clinical and biological characteristics of Figure 1A. (**A**) Distribution of PD-L1 protein expression levels on immune cells in each class (*p* = 0.0003 by the chi-squared test). (**B**) Objective response rate stratified by the three subgroups (*p* = 2.714 × 10^−5^ by the chi-squared test). (**C**) Comparison of the objective response rate between class 1 and class 3 in the GU subtype of the Lund classification (*p* = 0.046 by the two-tailed Fisher’s exact test). PD, progressive disease; SD, stable disease; PR, partial response; CR, complete response. (**D**) Distribution of subtypes of the Lund classification in each subgroup (*p* < 2.2 × 10^−16^ by the chi-squared test). UroA, urothelial-like A; GU, genomically unstable; Inf, infiltrated; UroB, urothelial-like B; SCCL, squamous cell carcinoma-like. (**E**) Distribution of subtypes of the TCGA classification in each subgroup (*p* < 4.6 × 10^−51^ by the two-tailed Fisher’s exact test). Lum-pap, luminal-papillary; Lum-inf, luminal-infiltrated; Lum, luminal; BS, basal squamous. (**F**) Reported tumor mutation burden (TMB) classified by the three subgroups (*p* = 1.83 × 10^−8^ by the two-sample *t*-test). (**G**) Reported TMB, classified by the three subgroups in the TCGA cohort (*p* = 0.012 by the two-sample *t*-test; class 2 vs. class 3). * *p* < 0.05, *** *p* < 0.001.

**Figure 3:**
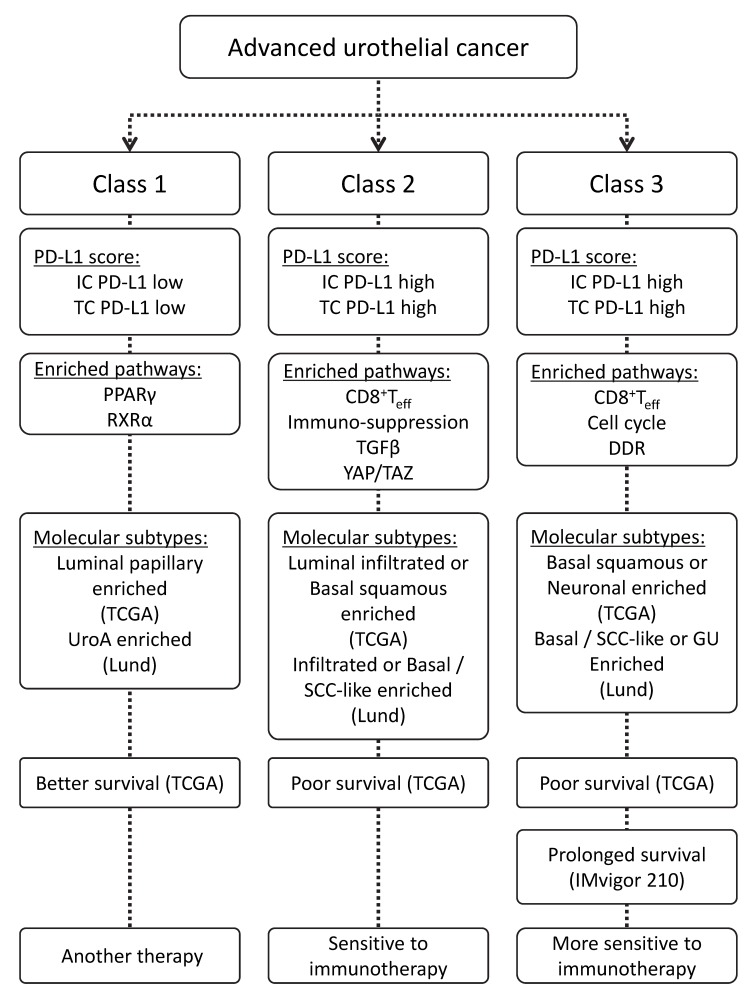
Schematic diagram of the characteristics of advanced urothelial cancer. T_eff_, T effector; DDR, DNA replication and DNA damage response; IC PD-L1, PD-L1 expression on immune cells; TC PD-L1, PD-L1 expression on tumor cell; SCC, squamous cell carcinoma; GU, genomically unstable.

PD-L1 protein expression on immune cells, which correlated with the activation of CD8^+^ T effector cells, was present in high scores over the classes 2 and 3 (Figure 2A). The results indicated that both PD-L1 protein expression and gene expression were largely related. When the objective response rate (ORR) was compared among these subgroups, class 3 exhibited a higher response rate than the other classes (Figure 2B). For a comparison with previously reported subtypes, we investigated the distribution of the Lund and TCGA subtypes in each subgroup [6,7]. The GU subtype of the Lund classification was mostly distributed in classes 1 and 3. When comparing the ORR of the GU subtypes across classes, interestingly, the complete response rate was significantly higher in class 3 than in class 1 (Figure 2C), implying that the activation of CD8^+^ T effector cell-related genes may play an important role in the response to ICI therapy beyond the GU subtype. The basal/SCC-like subtype of the Lund classification was divided into classes 2 and 3, whereas most of the patients with the infiltrated subtype were included in class 2 (Figure 2D). We also observed that the neuronal subtype, known as the most responsive subtype of the TCGA classification [10], was classified into class 3 (Figure 2E). These results indicate that patients who would be the most responsive to ICI therapy, including those with the neuronal, GU, or basal/SCC-like subtypes, could be re-stratified extensively by our classification. In addition, TMB was also significantly higher in class 3 than in other classes (Figure 2F). To validate the characteristics of the three subgroups, we applied our transcriptional patterns to other muscle-invasive bladder cancer patient cohorts. Similar gene expression patterns and TMB values were observed in the TCGA cohort (Figure S2 and Figure 2G). We also observed consistent biological characteristics in the Lund cohort (Figure S3). The gene expression patterns from the validation cohorts were also related to the activation of the cell cycle and the DDR and TGFβ and YAP/TAZ pathway genes, such as *FOXM1*, *TGFBR2*, and *CTGF* (Figure 1A, Figure 3 and Figure S2).

### 2.5. Schematic Diagram of the Characteristics of Advanced Urothelial Cancer

Based on our identified transcriptional patterns and the clinical outcomes of advanced urothelial bladder cancer patients, we constructed a schematic summary (Figure 3). In the TCGA cohort, the overall survival rate of the class 1 patients was significantly higher than that of the patients in classes 2 and 3. In addition, the IC PD-L1 and TC PD-L1 scores were relatively low in the class 1 patients. In contrast, for the patients in classes 2 and 3, the overall survival rate was significantly lower than that of the class 1 patients, and the IC PD-L1 and TC PD-L1 scores were relatively high. On the other hand, we suspect that the class 2 and class 3 patients had similar characteristics, but we observed a significant difference in biological pathways. In particular, a relative difference in the YAP/TAZ pathway has not yet been mentioned with other immunotherapies for advanced urothelial cancer. Taken together, these observations suggest that the high-risk patients in class 3 are the most likely to respond favorably to anti-PD-L1 treatment.

## 3. Discussion

Advanced urothelial cancer is clinically heterogeneous and exhibits poor outcomes. Using multiple bladder cancer patient cohorts, we carried out transcriptional profiling analyses, which identified a signature of distinct prognostic subtypes of advanced urothelial cancer. The signature showed therapeutic relevance in that those patients with enriched CD8^+^ T effector cell-related genes benefit from ICI therapy. Interestingly, among these individuals, patients with inactivation of the TGFβ and YAP/TAZ pathways and activation of the cell cycle and DDR were more responsive to ICI therapy than patients without these traits.

Recently, considerable effort has been devoted to elucidating the molecular characteristics of bladder cancer [6,7,8,9]. It has been reported that the GU subtype of the Lund classification and the neuronal subtype of the TCGA classification respond best to anti-PD-L1 treatment [10,11]. Despite these contributions, predicting clinically relevant patients responsive to ICI therapy remains a major challenge. We tried to directly compare the survival rates between the TCGA and the IMvigor 210 cohort. Through the results, beyond the previously known subtypes, we also tried to contribute to precisely selecting the patients who would be most responsive to treatment by introducing subtypes that reflect clinical characteristics and core biological pathways.

The most interesting finding of our study was the relative difference in the enriched biological pathways between our subtypes. In class 1, we confirmed that immune cell infiltration was controlled by activated PPARγ/RXRα, which inhibited host immune systems [12]. In recently updated data from TCGA, these patients showed enrichment of *FGFR3* mutations. Bladder cancer patients with *FGFR3* mutations have been associated with lower immune cell infiltration and lower TGFβ signals than patients without *FGFR3* mutations [13]. Patients with *FGFR* mutations or fusions may be less likely to have a response to immunotherapy than those without such alterations. The pan-FGFR inhibitor erdafitinib had a measurable benefit in patients with advanced urothelial carcinoma with *FGFR* alteration [16]. Therefore, we suggest that immunotherapy is not suitable for patients in class 1. In class 2, we identified an enrichment of CD8^+^ T effector cell-related genes. One of the most distinct characteristics was the co-activation of the TGFβ and YAP/TAZ pathways. Multiple cancer-associated signaling networks engage in regulatory crosstalk with the YAP/TAZ pathway, which has been reported to functionally interact with the TGFβ pathway. YAP/TAZ expression in immune cells, including T cells, B cells, and macrophages, regulates the differentiation and functionality of immune cells, which are important for tumor immunity [17]. Notably, CTGF, a major target gene that is associated with immune suppression and epithelial-mesenchymal transition, was differentially expressed. In class 3, we also identified enrichment of CD8^+^ T effector cells. However, the patients in class 3 showed inactivation of the TGFβ and YAP/TAZ pathways and activation of the cell cycle and the DDR. Cell cycle and DDR regulatory genes, which are significantly associated with TMB, play an important role in selecting patients with high response rates to ICI therapy.

However, the unclear relationship between DDR gene alterations and expression and response to immunotherapy remains a challenge. DDR gene alterations are independently associated with the response to PD-1/PD-L1 inhibitors in patients with advanced urothelial cancer [18]. Future studies using next-generation sequencing technologies will continue to uncover associations between mutation- or expression-based changes in tumor DNA repair pathway function and response to immunotherapy [19].

Through further analysis, we confirmed that patients with activated CD8^+^ T effector cells in class 2 and class 3 showed slightly prolonged survival after treatment with the PD-L1 inhibitor. In class 3, in particular, we identified that patients showed considerably prolonged survival after treatment. These patients also included a subset of patients with the GU subtype in the Lund classification and all the neuronal subtypes in the TCGA classification.

In conclusion, to effectively select patients who will respond to ICI therapy, we suggest that many aspects should be considered, including predefined subtypes, clinical characteristics, and core biological pathways. It is clear that the combined use of multiple markers can improve the performance of ICI therapy compared to a single marker. Our investigations will contribute to the development of predictive markers and therapeutic options.

## 4. Materials and Methods

### 4.1. Patients and Gene Expression Data

RNA-seq datasets from 348 patients with bladder cancer were obtained from the IMvigor 210 dataset [11]. Among the 348 patients, 298 patients who had received immunotherapy were used as the discovery cohort (The IMvigor 210 cohort, *n* = 298). Gene expression datasets from patients with bladder cancer from The Cancer Genome Atlas (TCGA, *n* = 407) and the Lund cohort (GSE83586, *n* = 307) were used as the validation cohorts [6,7]. Fragments per kilobase of transcript per million mapped reads (FPKM) values were calculated from sequence read count data in the IMvigor 210 dataset. All gene expression data were transformed to a log2 scale and normalized by quantile normalization. Clinical data were obtained from the supplementary information of the corresponding literature.

### 4.2. Gene Expression Analysis

For the IMvigor 210 cohort, we selected 2366 genes with FPKM values that were detected with confidence (FPKM > 1) and exhibited at least a two-fold difference relative to the median value in greater than 30% of the samples. To classify patients into three groups, we used a by-hierarchical clustering algorithm using the centered correlation coefficient as the measure of similarity and centroid linkage clustering. To develop the prediction model, we used Prediction Analysis for Microarrays (PAM) and 1659 selected genes (R-package: PAMR). To explore significantly enriched functions, we performed gene ontology (GO) enrichment analysis using the DAVID tool (http://david.ncifcrf.gov) with significance criteria (FDR < 0.01). To integrate previous gene sets with our signature, we standardized a pan-fibroblast TGFβ response signature (pan-F-TBRS) and T-cell-inflamed GEP score [11,14]. Hierarchical clustering analysis was conducted using Gene Cluster 3.0 and visualized using TreeView^TM^.

### 4.3. Statistical Analysis and Data Visualization

We estimated patient prognosis using Kaplan–Meier plots and the log-rank test. The significance of the distribution of subtypes and comparisons of objective responses were estimated using Fisher’s exact test. The significance of the distribution of IC PD-L1 protein expression levels was estimated using the chi-squared test. The reported tumor mutation burden (TMB) was estimated using the two-sample *t*-test. All statistical analyses were performed in the R 3.6.1 language environment (http://www.r-project.org).

### 4.4. Data Availability

IMvigor 210 data and clinical information were obtained from the IMvigor210CoreBiologies R package [11]. The Cancer Genome Atlas (TCGA) and the Lund datasets were obtained through Cancer Browser (https://xenabrowser.net) and Gene Expression Omnibus (GEO), respectively, with the accession number GSE83586.

## 5. Conclusions

We identified three molecular subtypes of advanced urothelial cancer that reflect clinical and biological features and consider the TCGA and Lund classifications. When comparing clinical and transcriptome data, patients with downregulation of the TGFβ and YAP/TAZ pathways and upregulation of the cell cycle, DNA replication and DNA damage response showed significantly prolonged survival. Because only a subset of patients benefits from immune checkpoint inhibitor therapy, our investigations will contribute to the development of predictive markers and therapeutic options.

## Supplementary Materials

The following are available online at https://www.mdpi.com/1422-0067/21/5/1850/s1, Figure S1: Hierarchical clustering analysis of gene expression data from the IMvigor 210 cohort. Three subgroups of patients and four distinct subsets of genes were revealed from unsupervised clustering analysis. The genes were grouped as G1, G2, G3, and G4. G1 was highly enriched in genes involved in the immune response and significantly highly expressed in classes 2 and 3. G2 was highly enriched in genes involved in angiogenesis, collagen fibril organization, and wound healing associated with the immunosuppressive reaction, and it was recently shown that this group attenuates the immune reaction towards the tumor via the TGFβ pathway. G3 was highly enriched in cell cycle-, histone-, or DNA repair-associated genes, implying that the high responsiveness to PD-L1 blockade of class 3 may be mediated by these genes. G4, predominantly expressed in class 1, was enriched in the metabolic process and FGFR3 pathway genes; Figure S2: Validation of the TCGA cohort (n=407). Heat map of the selected gene list associated with Figure 1. Samples are ordered according to the TCGA subtypes in each subgroup; Figure S3: Validation of the Lund cohort (n=307). Heat map of the selected gene set in the Lund cohort. Samples are ordered according to the three subgroups associated with Figure 1.
