# Supplementary material for: Transcriptional Profiling of Advanced Urothelial Cancer Predicts Prognosis and Response to Immunotherapy
Source: Int J Mol Sci. 2020 Mar 8;21(5):1850. doi: 10.3390/ijms21051850 (PMC7084828; doi:10.3390/ijms21051850)
Supplement: Supplementary file 1 [file ijms-21-01850-s001.pdf]

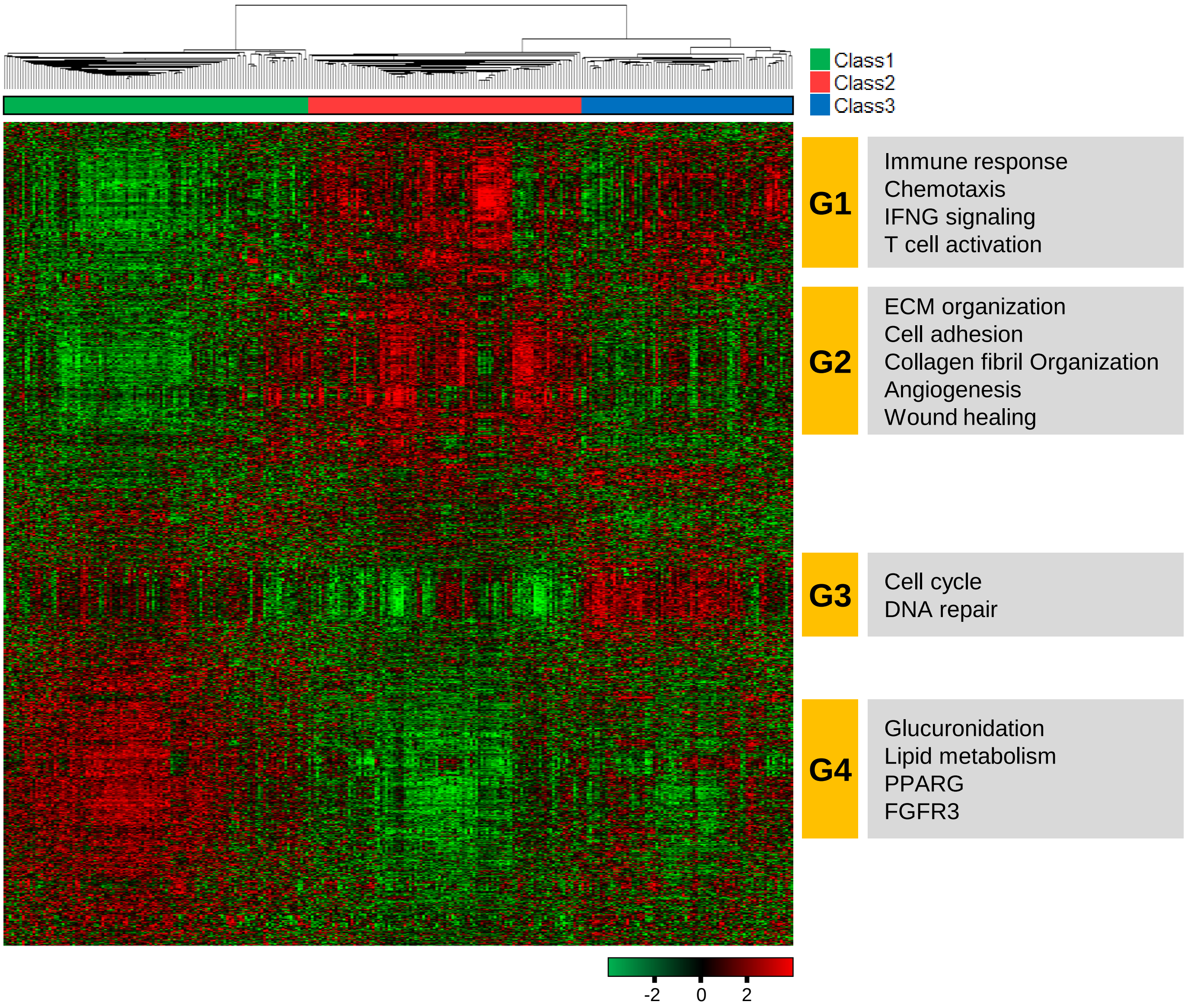

Supplementary Fig. 1 - Hierarchical clustering analysis of gene expression data from the IMvigor 210 cohort.

Three subgroups of patients and four distinct subsets of genes were revealed from unsupervised clustering analysis. The genes were grouped as G1, G2, G3, and G4. G1 was highly enriched in genes involved in the immune response and significantly highly expressed in classes 2 and 3. G2 was highly enriched in genes involved in angiogenesis, collagen fibril organization, and wound healing associated with the immunosuppressive reaction, and it was recently shown that this group attenuates the immune reaction towards the tumor via the TGFβ pathway. G3 was highly enriched in cell cycle-, histone-, or DNA repair-associated genes, implying that the high responsiveness to PD-L1 blockade of class 3 may be mediated by these genes. G4, predominantly expressed in class 1, was enriched in the metabolic process and FGFR3 pathway genes.

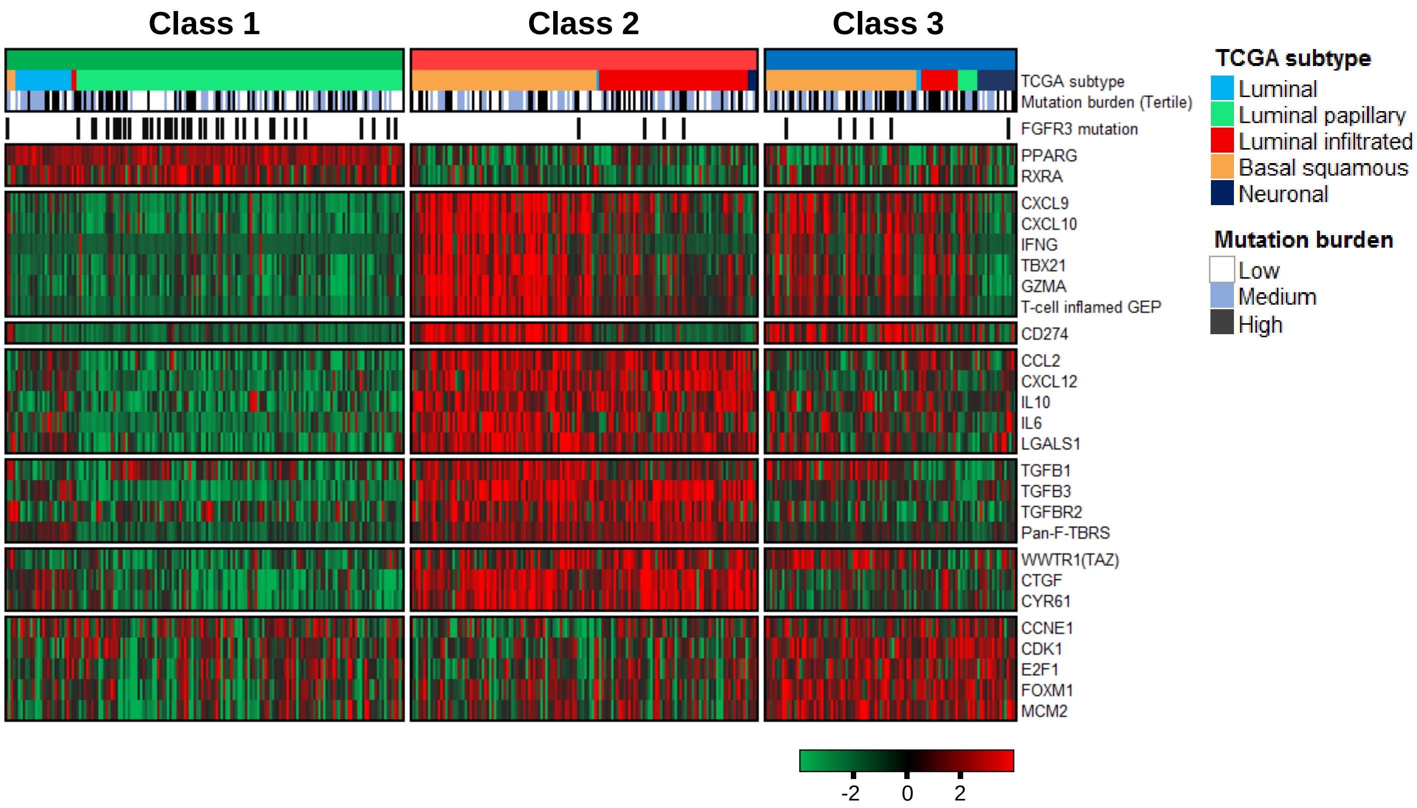

Supplementary Fig. 2 - Validation of the TCGA cohort (n=407). Heat map of the selected gene list associated with Fig. 1. Samples are ordered according to the TCGA subtypes in each subgroup.

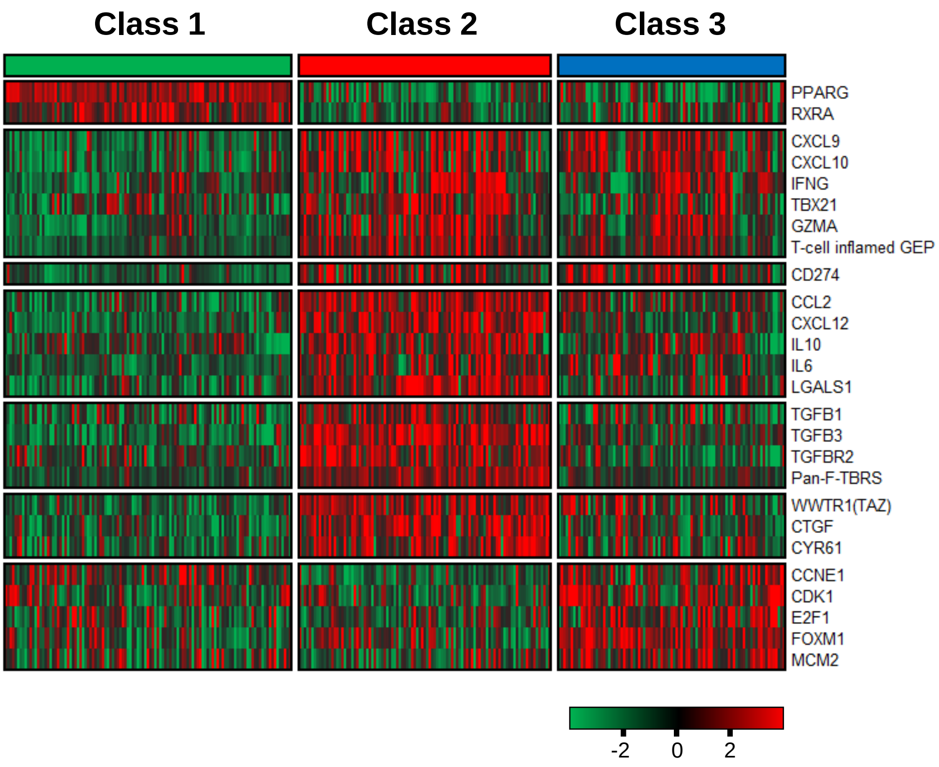

Supplementary Fig. 3 - Validation of the Lund cohort (n=307). Heat map of the selected gene set in the Lund cohort. Samples are ordered according to the three subgroups associated with Fig. 1.
